# Hantavirus Infection in the Republic of Georgia

**DOI:** 10.3201/eid1509.090617

**Published:** 2009-09

**Authors:** Tinatin Kuchuloria, Danielle V. Clark, Matthew J. Hepburn, Tengiz Tsertsvadze, Guillermo Pimentel, Paata Imnadze

**Affiliations:** Technology Management Company, Tbilisi, Georgia (T. Kuchuloria); Walter Reed Army Institute of Research, Silver Spring, Maryland, USA (D.V. Clark); United States Army Medical Research Institute of Infectious Diseases, Fort Detrick, Maryland, USA (M.J. Hepburn); Infectious Pathology, AIDS and Clinical Immunology S/P Center, Tbilisi (T. Tsertsvadze); Naval Medical Research Unit #3, Cairo, Egypt (G. Pimentel); National Center for Disease Control and Public Health, Tbilisi (P. Imnadze)

**Keywords:** Hantavirus, hantavirus infections, viruses, Georgia, acute febrile illness, dispatch

## Abstract

We describe a laboratory-confirmed case of hantavirus infection in the Republic of Georgia. Limited information is available about hantavirus infections in the Caucasus, although the infection has been reported throughout Europe and Russia. Increasing awareness and active disease surveillance contribute to our improved understanding of the geographic range of this pathogen.

Rodent-borne hantavirus infections causing hemorrhagic fever with renal syndrome (HFRS) occur throughout most of Europe and Russia. The pathogenic hantaviruses detected in Europe and Russia include Puumala, Dobrava, Saaremaa, Tula, Amur, and Hantaan viruses (*1**,**2*). Seroprevalence studies of hantavirus infection in Europe have shown prevalence rates as high as 6%–9% in Sweden, Estonia, and European Russia (*1*). The circulation of hantaviruses is specific predominantly to rodent species and thus correlates with the host range of the rodent reservoir. Several lineages of Dobrava virus circulate in Europe and Russia (*3**–**6*), and are named according to their rodent reservoirs: *Apodemus flavicollis* (Dobrava-Af), *A. agrarius* (Dobrava-Aa), and *A. ponticus* (Dobrava-Ap)*.* Dobrava-Aa is suspected to be the predominant strain in central Europe (*7*).

The clinical manifestations of hantavirus infection vary and depend largely on the strain of the infecting virus. Classic HFRS is characterized by fever, acute renal failure, hypotension, hemorrhage, and vascular leakage. Puumala virus typically induces a mild variant of HFRS (nephropathia epidemica) accompanied by high fever, headache, backache, and abdominal pain. Mild hemorrhagic manifestations, including conjunctival hemorrhage or petechiae on the trunk or palate, also may occur. Severe clinical manifestations are rare (<1% of patients) but can include acute renal failure, severe neurologic manifestations, and death (*8*). Dobrava-Aa may induce a similarly mild clinical course of illness to that of Puumala virus (*6*).

Information is scant about hantaviruses in the Republic of Georgia. Although reports of hantavirus infection from other countries in the surrounding area suggest that this pathogen circulates in the Caucasus, the incidence of these infections is unknown.

## The Patient

A 31-year-old male resident of Tbilisi, Georgia, sought treatment at the Infectious Pathology, AIDS and Clinical Immunology Center in early June 2008, six days after onset of illness. At that time, he was enrolled in an ongoing laboratory-based surveillance study of acute febrile illness (AFI). Initial clinical symptoms were fever (maximum axillary temperature 39°C), arthralgias and myalgias in the lower extremities, back pain, vomiting, and diffuse abdominal pain. Vital signs indicated a pulse rate of 87 bpm and blood pressure of 140/80 mm Hg. The physical examination noted mild hepatosplenomegaly and pharyngeal injection. The chest radiograph and ultrasound of the abdomen were normal. No preexisting illnesses were reported. Travel history included regular trips to Marneuli district (a rural district south of Tbilisi). No specific rodent exposures were noted, and no other travel was reported.

Laboratory findings were normal, with the exception of elevated band neutrophils (20%, reference range 1%–6%) and lymphocytosis (44%, reference range 19%–37%). C-reactive protein level was elevated (48.9 mg/mL, reference range <6 mg/mL), and decreased total protein (62 g/L, reference range 65–85 g/L) and albumin levels (20 g/L, reference range 35–50 g/L) were observed. Results of liver function tests and serum creatinine levels were within normal limits.

An elevated creatinine level (214 μmol/L, reference range 53–115 μmol/L) was noted on day 9 of illness, along with a decrease in urine output. Proteinuria (3 g/24 h), microscopic hematuria, and elevated blood pressure (160/90 mm Hg) were also observed. The patient was transferred to the nephrology department of the Institute of Urology in Tbilisi with a diagnosis of acute renal failure. Patient’s fluid input and output were closely regulated, and his condition improved gradually without dialysis.

Serum samples were tested for antibodies against a panel of pathogens, including *Salmonella enterica* serovar Typhi, *S. paratyphi* A and B, Epstein-Barr virus, *Brucella* spp., *Coxiella burnetti*, hantavirus, *Rickettsia* (spotted fever and scrub typhus groups), West Nile virus, tick-borne encephalitis virus, and *Leptospira* spp. All serologic test results were negative except for the test result for hantavirus. To corroborate hospital laboratory diagnostics, serum samples were also tested in a private laboratory in Germany by using Western blot assay (recomBlot Bunyavirus immunoglobulin [Ig]G/IgM; Mikrogen, Neuried, Germany) and immunofluorescence antibody (IFA) assay (Progen, Heidelberg, Germany). Results from the Western blot assay were negative for Puumala IgG and positive for Hantaan IgG and Dobrava IgM. The IFA result for Hantaan antibodies was positive at a reciprocal titer >2,048. The Naval Medical Research Unit No. 3 in Egypt conducted a hantavirus IgM ELISA (Focus Diagnostics, Cypress, CA, USA) on paired serum samples (acute-phase sample positive:negative ratio = 8.42; convalescent phase = 7.815, cut-off 1.10). Results of blood and *Leptospira* spp. cultures were negative. A renal biospy specimen obtained 10 days after disease onset showed acute tubular necrosis with mild-grade arteriolosclerosis (Figure).

**Figure:**
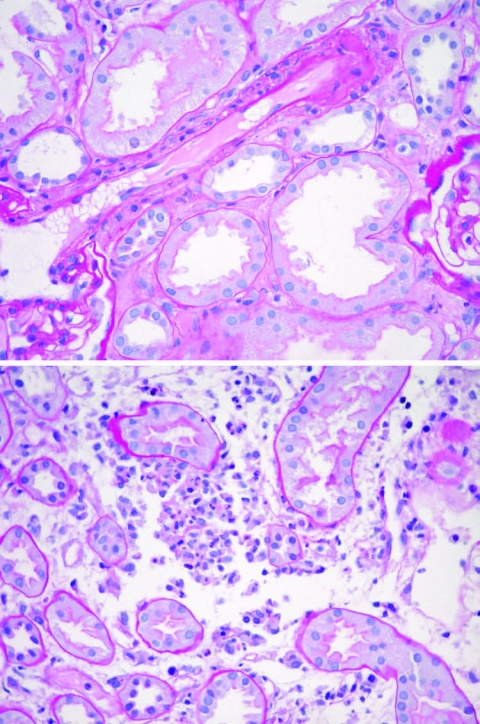
Acute tubular necrosis in a renal biopsy specimen of the patient. Magnification ×40.

On the 11th day of illness, the patient’s general condition started to improve, with increased urine output, resolution of abdominal pain, and normalization of blood pressure (120/70 mm Hg). No sequelae were reported at 1-month follow-up.

## Conclusions

The clinical presentation and results of serologic tests for this patient who had renal failure but no apparent hemorrhage were consistent with results previously described for hantavirus infection. Although the differential diagnosis included other infectious and noninfectious causes of renal failure including leptospirosis, ongoing surveillance of AFI in Georgia provided the serologic testing capability that enabled clinicians to identify a hantavirus infection as the cause of this illness characterized by high fever and evidence of renal dysfunction (increased serum creatinine, proteinuria, and hematuria).

The pathologic changes observed in hantavirus infection presumably result from increased vascular permeability, characterized clinically by elevated hematocrit and decreased serum protein levels. Damage to the vascular endothelium along with cytokine-induced renal tubular and interstitial pathologic changes may partially explain renal failure associated with hantavirus infection. Although the most commonly noted renal pathologic finding is acute interstitial nephritis, acute tubular cell necrosis is also described. A comparative study of Puumala and Dobrava renal pathology noted increased intensity and extent of medullary interstitial capillary injury, hemorrhages, and tubular necrosis among Dobrava virus-infected patients (*9*).

The strain of the infecting virus is unknown, but serologic results, along with the available information from surrounding countries, implicate Dobrava virus as the infecting strain. The relatively mild clinical symptoms of this patient suggest that a variant of Dobrava virus, or a closely related hantavirus strain that cross-reacts with Dobrava, may be the etiologic agent. Information about the circulating virus strains, clinical manifestation, rodent hosts, and disease prevalence are lacking for the Caucasus. Further studies are needed on potential rodent hosts and characterization of circulating strains.
